# Supplementary material for: Attrition Rates in Multiple Myeloma Treatment under Real World Conditions—An Analysis from the Austrian Myeloma Registry (AMR)
Source: Cancers (Basel). 2023 Feb 2;15(3):962. doi: 10.3390/cancers15030962 (PMC9913775; doi:10.3390/cancers15030962)
Supplement: Supplementary file 1 [file cancers-15-00962-s001.zip › cancers-2153531-supplementary.pdf]

Table S1: DIFFERENCES IN ATTRITION RATE IN RESPECT OF STEM-CELL TRANSPLANTATION

SCT NOT ELEGIBLE

LoT number	Number of patients	Treatment completed in numbers	AR	Subsequent LOT	Median Age
**1st LOT**	**NTE**<br>56.8% (288 n)	72.9%<br>220 n.	21.9 %<br>63 n.	39 %<br>107 n.	78 yrs.<br>71-82 yrs. IQRs
	**TE**<br>43.2% (219n)	65.3%<br>160 n.	16 %<br>35 n.	38.4 %<br>84 n.	64 yrs.<br>56-70 yrs. IQRs
**2nd LOT**	**NTE**<br>83.2% (159 n)	70.4%<br>123 n.	19.5 %<br>31 n.	47.8 %<br>76 n.	74 yrs.<br>65-81 yrs. IQRs
	**TE**<br>16.8% (32 n)	59.4 %<br>19 n.	18.8 %<br>6 n.	28.2%<br>9	63 yrs.<br>59-71 yrs. IQRs
**3rd LOT**	**NTE**<br>88.2% (75 n)	72 %<br>54 n.	24 %<br>18 n.	44 %<br>33 n.	74 yrs.<br>65-80 yrs. IQRs
	**TE**<br>11.8% (10 n)	80%<br>8 n.	20 %<br>2 n.	40 %<br>4 n.	63 yrs.<br>59-71 yrs. IQRs

Table S1: The table shows the difference between transplant eligible (TE) patients and patients ineligible for transplant (TE) in respect of attrition rate (AR) presented in percentage (%) and number (n). The number of patients who completed the line of therapy (LoT) is given for LoT 1-3. The amount of patient continuing with the subsequent LoT is illustrated. Median age in years (yrs.) with interquartile range (IQRs) is presented for each group. After 3rd, LoT the number of patients is too limited to draw definite conclusions.
